# 3D pelvimetry and biometric measurements: a surgical perspective for colorectal resections

**DOI:** 10.1007/s00384-020-03802-9

**Published:** 2020-11-23

**Authors:** Laura Lorenzon, Fabiano Bini, Federica Landolfi, Serena Quinzi, Genoveffa Balducci, Franco Marinozzi, Alberto Biondi, Roberto Persiani, Domenico D’Ugo, Flavio Tirelli, Elsa Iannicelli

**Affiliations:** 1 General Surgery Unit, Fondazione Policlinico Universitario Agostino Gemelli IRCCS, Catholic University, Rome, Italy; 2 Surgical and Medical Department of Translational Medicine, Sant’Andrea Hospital, Faculty of Medicine and Psychology, “Sapienza” University of Rome, 00185 Rome, RM Italy; 3 Department of Mechanical and Aerospace Engineering, “Sapienza” University of Rome, via Eudossiana 18, 00184 Rome, Italy

**Keywords:** Rectal cancer, TaTME, Total mesorectal excision, 3D imaging

## Abstract

**Purpose:**

Male sex, high BMI, narrow pelvis, and bulky mesorectum were acknowledged as clinical variables correlated with a difficult pelvic dissection in colorectal surgery. This paper aimed at comparing pelvic biometric measurements in female and male patients and at providing a perspective on how pelvimetry segmentation may help in visualizing mesorectal distribution.

**Methods:**

A 3D software was used for segmentation of DICOM data of consecutive patients aged 60 years, who underwent elective abdominal CT scan. The following measurements were estimated: pelvic inlet, outlet, and depth; pubic tubercle height; distances from the promontory to the coccyx and to S3/S4; distance from S3/S4 to coccyx’s tip; ischial spines distance; pelvic tilt; offset angle; pelvic inlet angle; angle between the inlet/sacral promontory/coccyx; angle between the promontory/coccyx/pelvic outlet; S3 angle; and pelvic inlet to pelvic depth ratio. The measurements were compared in males and females using statistical analyses.

**Results:**

Two-hundred patients (M/F 1:1) were analyzed. Out of 21 pelvimetry measurements, 19 of them documented a significant mean difference between groups. Specifically, female patients had a significantly wider pelvic inlet and outlet but a shorter pelvic depth, and promontory/sacral/coccyx distances, resulting in an augmented inlet/depth ratio when comparing with males (*p* < 0.0001). The sole exceptions were the straight conjugate (*p* = 0.06) and S3 angle (*p =* 0.17). 3D segmentation provided a perspective of the mesorectum distribution according to the pelvic shape.

**Conclusion:**

Significant differences in the structure of pelvis exist in males and females. Surgeons must be aware of the pelvic shape when approaching the rectum.

**Supplementary Information:**

The online version contains supplementary material available at 10.1007/s00384-020-03802-9.

## Introduction

Over the last few years, the surgical treatment of rectal cancer evolved at a rapid pace. Implementation of technologies led to the introduction of mini-invasive techniques, but their widespread clinical adoption was impaired due to several reasons, including costs (i.e. robotics procedure), and technical difficulties (i.e. limitation of the surgical instruments and articulation of the laparoscopic devices) [1].

If availability, limitations, and costs of surgical devices could represent issues, also patients’ and tumour characteristics influenced surgical choices. Male sex, high BMI, narrow pelvis, and bulky mesorectum were all acknowledged in literature as the clinical variables correlated with a difficult pelvic dissection and incomplete mesorectal excision or positive distal/radial margins [2].

Nevertheless, a novel approach is currently emerging which combines the benefits of mini-invasive surgery with the principles of surgical oncology and total mesorectal excision (TME). Trans-anal TME (TaTME) procedures gained interest in relation to a reduced conversion rate and longer distal resection margins comparing laparoscopic trans-abdominal low rectal resections [3]. Along with its benefits, few pitfalls started to emerge when approaching the rectum bottom-up, since the surgical field is demanding and performing a mesorectal dissection could be difficult in relation to the challenging anatomy [4, 5]. New adverse events such as nerve, vessel, and urethral injuries have been described and several authors reported new tips for dissection and preservation of the correct planes [4, 6]. Of note, up to 40% of current TaTME literature focuses on its complications (Supplement Figure 1).

Though the notion that the surgical procedure is easier in a wider pelvis is a well-recognized matter among colorectal surgeons, the anatomy and its biometric measurements are gaining interest; indeed, a complete understanding of the pelvis anatomy is essential for colorectal surgeons and it can provide the basis for a sharp dissection with curative intent during the resection of rectal cancers [7].

Pelvimetry, measurement of pelvic bony dimensions, has been performed for over half a century, in attempts to predict cephalo-pelvic disproportion prior to labour. 2D and 3D pelvimetry reconstructions, both using CT scans or MRI [8, 9], are gaining a momentum in this field, with the main outcomes of predicting surgical difficulties, or achieving a complete mesorectal plane, negative circumferential margins, or a sphincter-saving procedure [9–11].

Pelvimetry has been also applied to modern radiology to investigate differences in pelvic diameters of female and male patients undergoing rectal surgery procedures, even if with contradicting results [8, 12].

The aim of this study was to investigate differences between female and male patients of the pelvic biometric measurements obtained using 3D segmentation of CT scans and to provide a perspective on how pelvimetry may help in visualizing mesorectum distribution.

## Materials and methods

All consecutive patients born in 1955, 1956, and 1957 who underwent an elective or emergency abdominal CT scan with or without contrast enhancement at Sant’Andrea Hospital of Rome respectively during 2015, 2016, and 2017, independently from the indication, were considered eligible and retrospectively reviewed. The choice of 60-year-old patients was made consistently with the aim of this study which had the objective to evaluate pelvimetry in a typical adult, asymptomatic, and at average risk population for colorectal cancer [13]. Patients who had pelvic and/or femur fractures, those with fixation devices, or those with incomplete pelvic bone acquisitions were excluded. Of note, the reason to include emergencies and elective procedures with or without contrast was motivated by the fact that soft tissues were not the objective of 3D segmentation, and thus, exclusion criteria were limited to those conditions impairing imaging modelling.

Computed tomography scans were performed with multi-detector CT equipment, GE Light Speed 16. The following technical parameters were used: 120 kV; 120 to 180 mA; gantry rotation time, 0.5 s; beam collimation, 16 × 1.25 mm; beam pitch, 1; and reconstruction thickness, 2.5 mm.

### Pelvimetry

DICOM data from selected patients were analyzed using 3D Slicers Software, version 4.5.0-1. From the axial plane view, each slice was processed by means of automatic segmentation algorithm level tracing effect, available in the Editor menu. Starting from an initial pixel (termed seed), the algorithm performs a comparison between adjacent pixels (neighbours) and draws closed lines around pixels having the same grey level as the seed. CT images consist in maps of the *μ* (*x*, *y*) absorption coefficients of the different materials and this allowed to easily implement automatic segmentation tools since the bone tissue offers the greatest contrast with respect to the background material; therefore, the bone structures are the most clearly visible and identifiable also in terms of grey levels [14]. Once all the slides were processed, the related 3D model was generated. The real pelvic surface was digitalized by approximating it to a certain number of polygons (polygonal mesh). In order to reduce the digitalization effects without significant detail loss and to provide a more accurate view, smoothing operations were performed on the 3D model. After the smoothing and filtering operations, the 3D model volume was reduced to less than 1%; therefore, the model geometry was not significantly impacted by the filtering process. The filtered 3D model was compared with the original one, and then 18 fiducial markers were applied on the points of interest of the 3D model. Since the software is able to store the spatial coordinates of each marker, it was possible to evaluate relevant measurement and pelvic angles (Supplement Figure 2).

Patients were categorized according to sex and the following pelvimetry measurements were estimated [12]:

1. *Pelvic inlet*: obstetric conjugate, true conjugate, and diagonal conjugate (lines from the superior, middle, and inferior pubic symphysis to the sacral promontory); anatomical transverse diameter (farthest distance between iliopectineal lines); and oblique diameter (from the sacroiliac joint to the iliopectineal eminence)
2. *Pelvic outlet*: straight conjugate (from the lower border of the pubic symphysis to the tip of coccyx); median conjugate (from the lower border of the pubic symphysis to the lower border of the sacrum); bis-ischiatic diameter
3. *Other measurements*: pubic tubercle height, distance from the sacral promontory to the coccyx; pelvic depth, a line taken from the midpoint of the pelvic inlet to the coccyx; distance from the sacral promontory to S3/S4 intervertebral disc; distance from S3/S4 intervertebral disc to the tip of the coccyx; and ischial spines distance
4. *Angles*: pelvic tilt (the angle between the vertical plane and the line that travels through the midpoint of the sacral platform toward the centre of the femoral heads); the offset angle (*α*), the pelvic inlet angle (*β*), the angle between the inlet, sacral promontory and coccyx (*χ*), the angle between the promontory, the coccyx and pelvic outlet (*δ*), the angle at S3 (*ε*), and the pelvic inlet to pelvic depth ratio

The bony structure of the pelvis was then 3D modelled to simulate the surgical view when approaching the pelvis trans-abdominally or trans-perineally. Also, to evaluate how these measures may impact mesorectum distribution, the most representative imaging scans of patients undergoing TaTME procedure for rectal cancer at the Fondazione Policlinico Universitario A. Gemelli in 2019 in Rome were used to provide the surgical perspective concerning mesorectal distribution.

### Statistics

A priori power analysis computation was performed to estimate the sample size to test the difference between the subgroups using *t* tests (difference between two independent means) using the following parameters: input: two tails; effect size *d* = 0.4; *α* err prob = 0.05; power (1 − *β* err prob) = 0.80; allocation ratio *N*2/*N*1 = 1. Results obtained were the following: non-centrality parameter *δ* = 2.8284271; critical *t* = 1.9720175; Df = 198, estimating two groups of 100 patients for an actual power of 0.8036475.

Measurements obtained by pelvimetry calculations were recorded using millimetres and records were de-identified in a database using a consecutive number in each sub-group. All the tests were performed two-tailed and a *p* value < 0.05 was considered statistically significant. Statistical analyses were obtained using MedCalc for Windows, version 10.2.0.0, and G*Power software version 3.1.2.

## Results

### Study population and pelvimetry

Over the study period, 284 patients were considered eligible for the 3D segmentation based on CT scans (100 patients born in 1955 who underwent imaging in 2015; 66 elective procedures, 30 males and 33 females, and 37 emergency, 28 males and 9 females; 81 patients born in 1956 who underwent imaging in 2016: 58 elective procedures, 29 males and 29 females, and 46 emergency, 28 males and 18 females; 103 patients born in 1957 who underwent imaging in 2017: 57 elective, 29 males and 28 females, and 46 emergency, 28 males and 18 females). The CT scans were reviewed retrospectively according to inclusion/exclusion criteria and revisions stopped when the accrual was achieved to select two groups of 100 female and 100 male patients.

Pelvimetry estimations were performed upon 3D reconstructions and results are presented in Table 1.

**Table 1:** Pelvimetry measurements in female and male patients

	Female patients, *N =* 100	Male patients, *N =* 100	*p* value
Pelvic inlet			
Obstetric conjugate (mm)			
Mean	126.2	119.4	
SD	8.6	9.9	4.36544E−07
True conjugate (mm)			
Mean	122.1	114.0	
SD	8.8	10.0	4.95965E−09
Diagonal conjugate (mm)			
Mean	131.8	127.9	
SD	8.8	10.3	0.004435735
Transverse diameter (mm)			
Mean	133.8	128.2	
SD	7.7	8.0	9.69001E−07
Oblique diameter (mm)			
Mean	130.0	125.7	
SD	7.2	6.3	1.01799E−05
Pelvic outlet			
Straight conjugate (mm)			
Mean	99.9	97.4	
SD	10.6	8.4	0.059169229
Median conjugate (mm)			
Mean	112.3	109.7	
SD	9.9	8.4	0.044227539
Bis-ischiatic diameter (mm)			
Mean	118.0	102.2	
SD	10.1	10.4	2.42427E−22
Other measurements			
Pubic tubercle height (mm)			
Mean	30.7	34.8	
SD	3.7	4.0	8.51241E−13
Promontory to coccyx (mm)			
Mean	119.0	128.7	
SD	12.3	11.8	4.2985E−08
Pelvic depth (mm)			
Mean	102.8	111.5	
SD	9.8	8.8	2.6971E−10
Sacral promontory to S3/S4 (mm)			
Mean	77.2	80.6	
SD	6.7	7.8	0.001366554
S3/S4 to coccyx (mm)			
Mean	63.4	69.6	
SD	7.7	8.6	1.90718E−07
Ischial spines distance (mm)			
Mean	116.7	100.5	
SD	9.3	8.6	8.08162E−28
Angles			
Pelvic tilt (mm)			
Mean	64.7	61.0	
SD	7.7	9.0	0.001978057
Offset α (mm)			
Mean	32.1	27.5	
SD	3.5	3.2	9.90042E−19
Pelvic inlet β (mm)			
Mean	47.1	52.9	
SD	8.2	8.1	1.02298E−06
Angle *χ* (mm)			
Mean	59.0	60.1	
SD	7.1	5.9	0.248535073
Angle δ (mm)			
Mean	72.7	67.6	
SD	8.9	6.9	7.32095E−06
Angle ε (mm)			
Mean	116.4	118.5	
SD	11.8	10.0	0.17291095
Pelvic inlet to pelvic depth ratio (mm)			
Mean	1.2	1.1	
SD	0.1	0.1	8.26754E−16

**Fig. 1:**
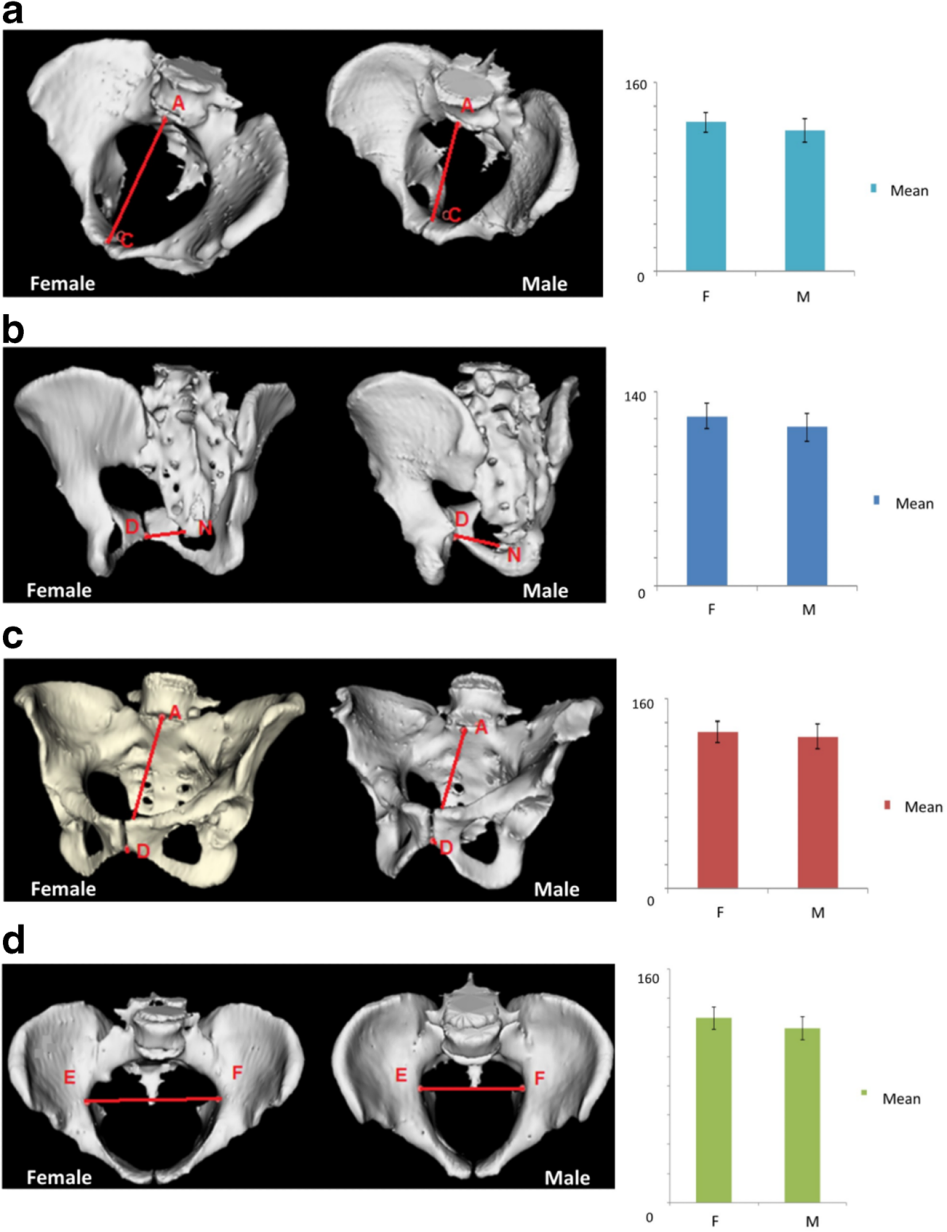
Pelvic inlet measurements in female vs male patients. **a** Obstetric conjugate, two representative patients, and graph bars showing mean difference in the 2 groups. **b** True conjugate, two representative patients, and graph bars showing mean difference in the 2 groups. **c** Diagonal conjugate, two representative patients, and graph bars showing mean difference in the 2 groups. **d** Transverse diameter, two representative patients, and graph bars showing mean difference in the 2 groups

**Fig. 2:**
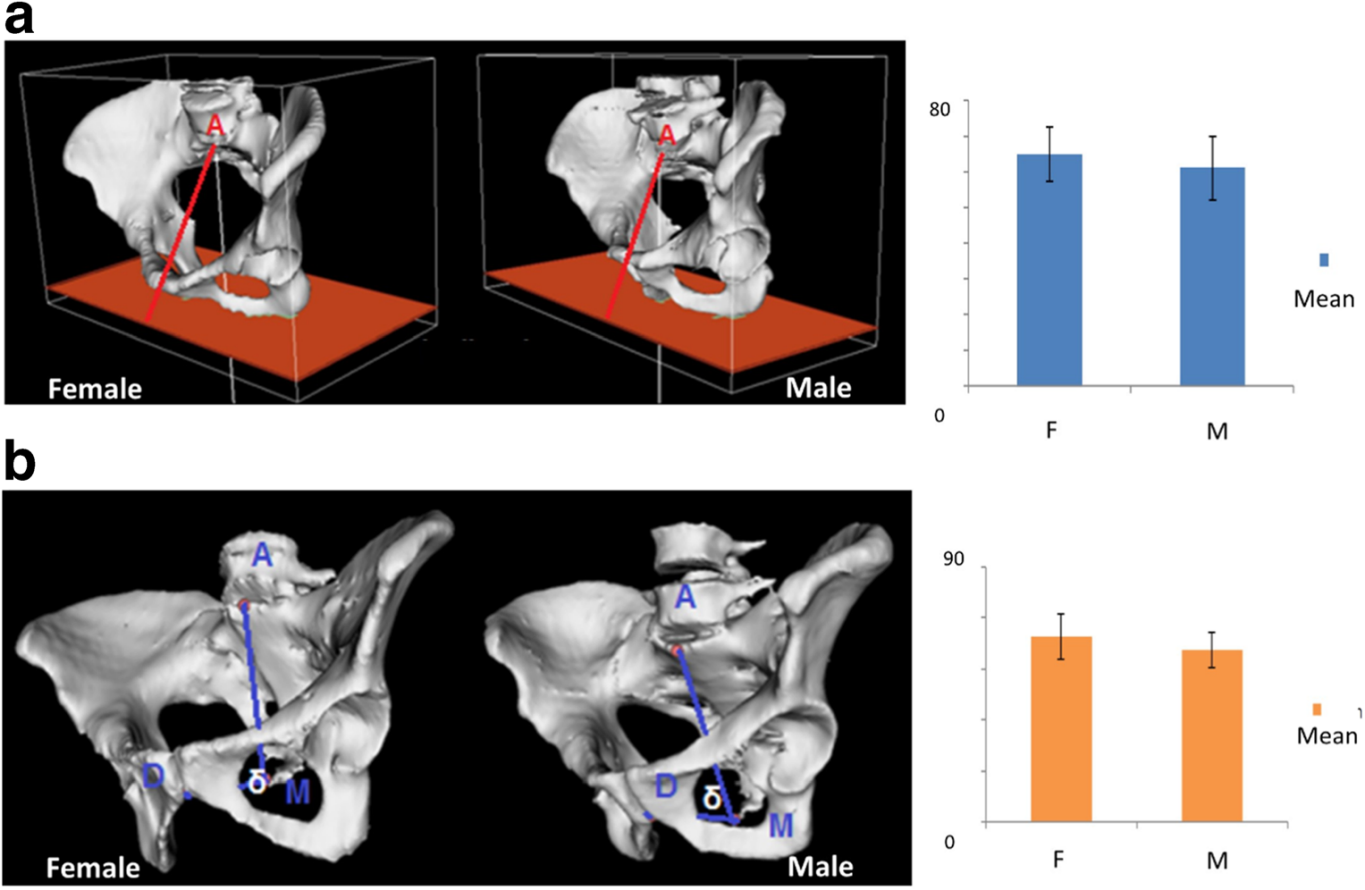
Pelvic angles in female vs male patients. **a** Pelvic tilt, two representative patients, and graph bars showing mean difference in the 2 groups. **b** Angle *δ*, two representative patients, and graph bars showing mean difference in the 2 groups

**Fig. 3:**
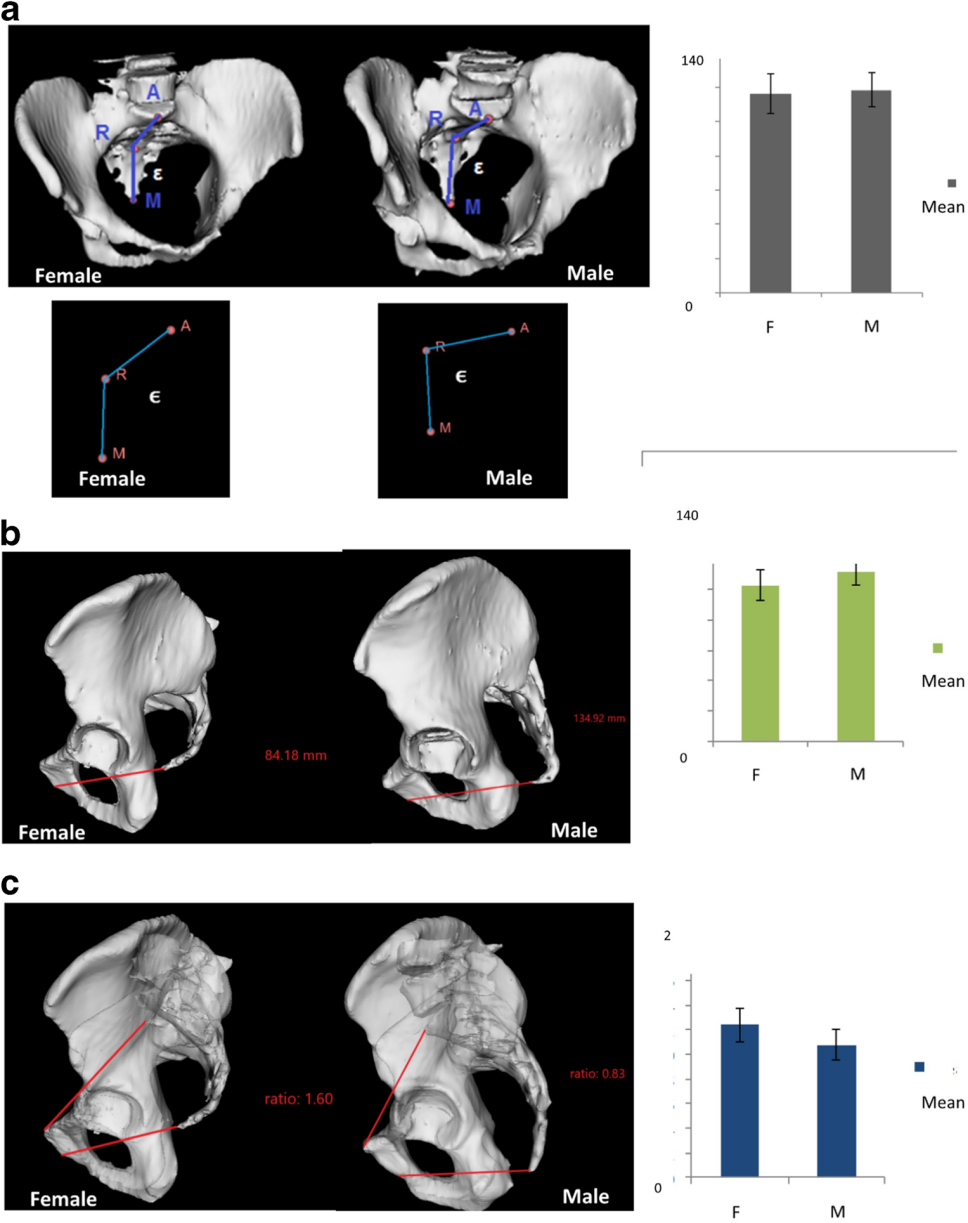
Pelvic narrowness in female vs male patients. **a** Angle *ε*, two representative patients (3D pelvimetry plus vectors representations), and graph bars showing mean difference in the 2 groups. **b** Pelvic depth, two representative patients, and graph bars showing mean difference in the 2 groups. **c** Pelvic inlet to pelvic depth ratio, two representative patients, and graph bars showing mean difference in the 2 groups

Out of 21 pelvimetry measurements, 19 of them documented a significant mean difference between the female and male population. Specifically, pelvic inlet was significantly wider in female vs male patients (Fig. 1). Also, pelvic outlet documented larger diameters for median conjugate and the bis-ischiatic diameter, with a borderline difference for the straight conjugate. Other measurements and pelvic angles documented dissimilarities of statistical value, with the sole exception of the angle *ε* (Figs. 2 and 3).

The narrowness of the pelvis results from the combination of the pelvic inlet, the pelvic depth, and their ratio; in a narrow pelvis, an acute angle of the sacrum *ε* could represent almost a perpendicular floor if the dissection is performed from the abdomen, and a vertical one when performed bottom-up (Fig. 3).

### Surgical perspective

Supplement Video 1 highlights how the bony structure of the pelvis may influence the surgical view when the pelvis is approached from the abdomen or from the perineum in relation to the pelvic inlet, outlet, and angles; in particular, angle *ε* could be a hallmark of difficulty when approaching the rectum bottom-up.


3D segmentation of the pelvis based on CT scans showing a patient with an angulated sacrum (left panel) vs a very flat sacrum (right panel). Coccyx is marked in red. Pelvis are shown in axial, frontal, sagittal view and also rotated to simulate a supine position and a trans-abdominal approach. (MP4 18,630 kb)

**Fig. 4:**
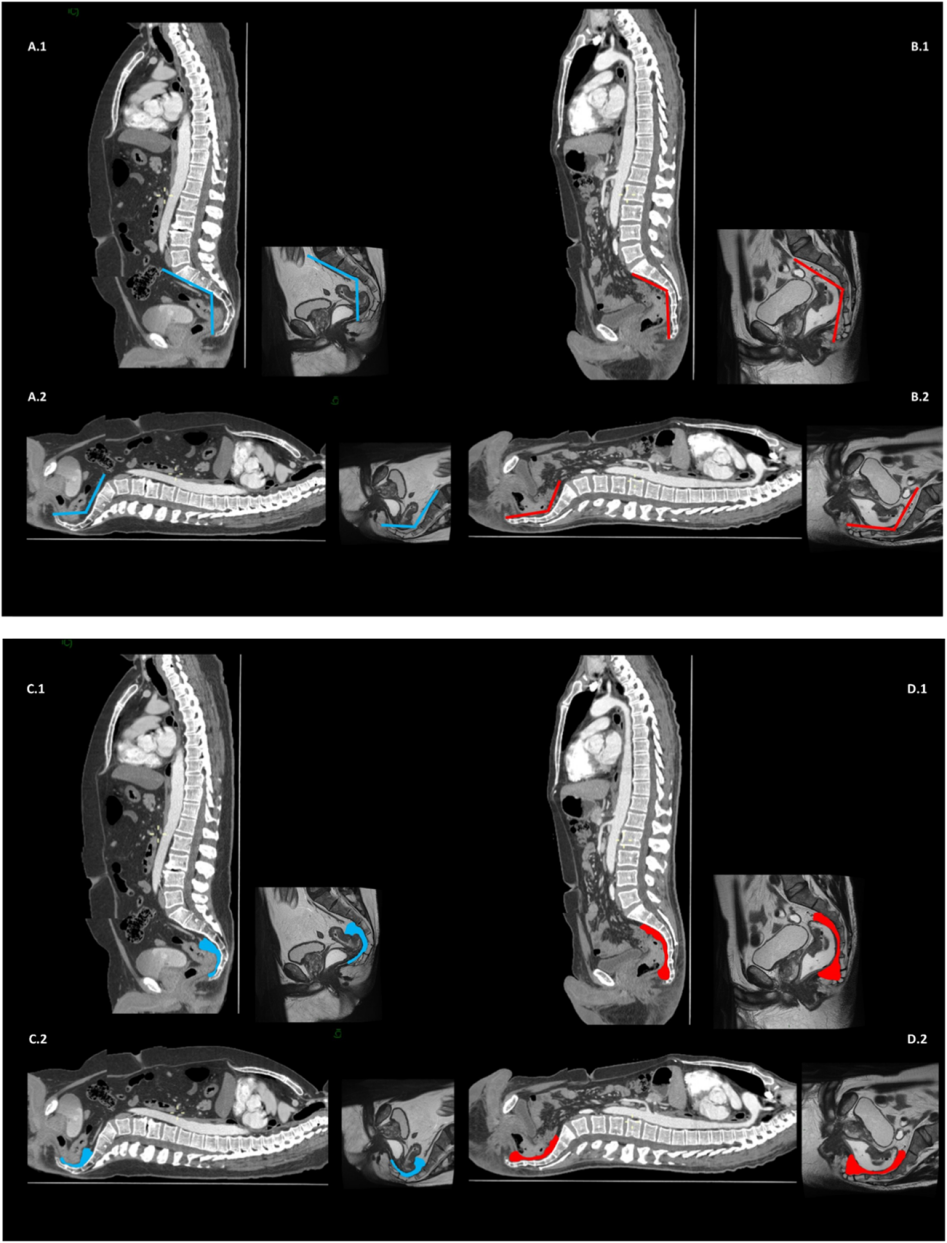
CT and MRI scans in a patient with rectal cancer documenting angle *ε* and posterior mesorectum. A1. Sagittal plane in a male patient with an acute angle *ε* represented by vectors on CT and MRI scans. A2. Same images showing difference when ideally approaching the rectum bottom-up. B1. Sagittal plane in a male patient with an obtuse angle *ε* represented by vectors on CT and MRI scans. B2. Same images showing difference when ideally approaching the rectum bottom-up. C1. Same patient presented in A1 and A2 with acute angle *ε*, highlighting posterior mesorectum, and C2 the difference when ideally approaching the rectum bottom-up. D1. Same patient presented in B1 and B2 with obtuse angle *ε*, highlighting posterior mesorectum, and D2 the difference when ideally approaching the rectum bottom-up

Another example of how these measurements may impact the morphology of posterior mesorectum is shown in Fig. 4: remarkably, the posterior/lower mesorectum was very thin in the first patient (narrow pelvis with acute angle *ε*) and well represented in the second one (wider pelvis with obtuse angle *ε*). Both these male patients underwent TaTME procedures for colorectal cancers. Indeed, a pelvimetry study may help in defining the burden of dissection, especially when considering the posterior mesorectum (Supplement Video 2).

3D segmentation and virtual prototyping based on CT and MRI scans of a female patient with rectal cancer. Scans are marked with areas for segmentation. A virtual model based on the segmentation is obtained and presented in sagittal and axial rotations. (MP4 40,379 kb)

## Discussion

The recent advantages made in colorectal surgery forced surgeons to change perspective in particular when approaching the anatomy of the rectum “bottom-up”. Colorectal surgeons must be aware of the challenges of developing a safe and correct surgical plane in line with the principles of surgical oncology. It is of particular interest the word of cautious used by Philip Quirke commenting on the St. Galle consensus on the safe implementation of TaTME. In his view, “the anatomy is difficult as posteriorly there are complex changes in the angulation of the mesorectal fascial plane and the mesorectum is initially a thin fatty layer. Anteriorly there is very little mesorectum and the anterior surgical margin is in juxtaposition to the urethra”. Also, tumour location is a very important factor, since lower tumours can generate worse surgical planes [15]. The perineal distances and trans-anal angles could be very different in female and male patients. Indeed, the female pelvis tends to be broader, with less prominent ischial spines, whereas in males, the pelvis has usually a longer, more curved sacrum, and a narrower sub-pubic arch, although literature relies on historical publications [16]. In a recent anatomic study, it was documented that the anterior perineum (mean distance between vagina/scrotum and anal verge) is significantly shorter in women comparing men. On the opposite, the rectoperineal angle (measured using a proctoscope) is significantly wider in the female population comparing men (clinical anatomy). If this applies for a proctoscope, it would be consistent to find a difference also when a flexible or rigid trans-anal platform is placed and used in procedures where dissection is based on geometrical tips (the so-called O and triangle dissection planes) [5] and on a dynamic anatomic distortion made by the pneumorectum [5].

Several authors correlated the anatomical variables with surgical results, for open, robotics, laparoscopic, and TaTME dissections [8, 17–24]. In a small series of patients who underwent open surgery for low rectal tumours, no significant differences were detected between the pelvis depth of females and males. Statistical analyses showed that the body mass index, tumour location, and several pelvimetry parameters, including pelvic inlet, pelvic outlet, height of the pubic symphysis, the sacrococcygeal distance, sacrococcygeal-pubic angle, and diameter of the upper pubis to the coccyx, significantly affected the operative time, while the tumour’s diameter correlated with blood loss. Interestingly, in this study, although patients’ clinico-pathological parameters seemed to predict difficulty in low anterior resection, the pelvic anatomical parameters appeared to correlate with variation in abdomino-perineal resection. According to authors’ conclusion, the intra-operative difficulty is likely to increase in deeper and narrower pelvises, or in those with greater sacrococcygeal curvature, consistently with the present perspective [20]. Other studies disclosed no significant correlations between pelvic dimensions and operative time in laparoscopic rectal resections, although results were based on a very small cohort of 50 patients [8]. These findings were however not confirmed by others, who documented that a less acutely curved sacrum and a larger sagittal pelvic outlet are significantly correlated with longer operations, especially in a pelvis with a narrow inter-tuberous distance [22]. Consistently, also another larger series of more than 200 patients confirmed that the shape of the pelvic inlet may be useful for predicting the operative time [18]. It should be acknowledged that pelvic bones, although have a key role in determining the shape of the pelvis, may not be sufficient in predicting surgical difficulties and oncologic results: indeed, another study pointed that it is the relative dimensions of the tumour within the pelvis which influence the local recurrence and overall survival rates of patients who underwent open surgery after neo-adjuvant treatment for primary rectal cancer [19]. Another study evaluated the mesorectal fat area calculated at the level of the tip of the ischial spines, in patients undergoing laparoscopic and robotic surgery: according to these researches, a larger mesorectal fat area was associated with longer operative time of the pelvic phase [20]. More recently, an international collaborative study elaborated a predictive score for operative (time) and post-operative results (hospitalization); this score was calculated based on body mass index (> 30), inter-spinous distance < 96.4 mm, ymrT stage ≥ T3b, and male sex, and demonstrated a high accuracy [23, 24]. Also, in a relatively large cohort of 121 rectal cancer patients undergoing mini-invasive treatment, multivariable analysis indicated that high-grade surgical difficulty was associated with a BMI > 25, a tumour size > 4.5 cm, an anorectal angle greater than 123°, and pelvic outlet less than 82.7 mm. All of these features were used to develop a scoring model to predict surgical difficulty. The anastomotic leakage rate was 53% in the high-risk group vs 9.6% in the low-grade group, and the former had also a significantly higher local recurrence rate comparing the others [25]. This trend was confirmed by a recent meta-analysis that highlighted how bony pelvic measurements may predict surgical difficulty during TME; however, the use of different indicators limits comparison between studies [26].

The bony architecture of pelvis, and in particular the distance between the inter-obturator foramina line and inferior pubic symphysis, the pubic arch angle, pubic ramus width, and pubic symphysis length were investigated also in urologic studies since they may potentially affect surgical procedures such as sub-urethral sling [27]. As far as it concerns specifically nerval injuries in the male population, the study of pelvimetric dimensions did not impact the likelihood of performing a nerve-sparing procedure in patients undergoing radical retropubic prostatectomy [28].

Although these kinds of modelling could be futuristic and not of clinical use for nowadays practice, it is possible that they will be implemented in a short future, in order to help surgeons in the understanding of surgical planes, if required, or implement a trans-abdominal procedure. Consistently with this line of research, a model for the 3D imaging and preoperative planning of a TaTME has been also proposed [17]. Real-time stereotactic navigation for TaTME has been reported to be feasible in small pilot series using laparoscopy, and currently, its feasibility in robotics in combination with indocyanine green fluorescence has been explored to identify structures [29], and the inclusion of 3D pelvimetry may add an additional benefit.

Despite the advantages, TaTME procedures have been also highly criticized for the long-term results reported by some authors, in particular with regard to a higher rate of local recurrence: one example is the Norwegian moratorium by the Norwegian Colorectal Cancer Group [30]. Thus, large international studies are highly advocated. In keeping with this, a large prospective, observational, case-matched, four-cohort, multicentre trial is currently ongoing and designed to study TME using open laparotomy, laparoscopy, robot-assisted surgery, or trans-anal surgery in 1300 high-surgical-risk patients (BMI > 30, involvement of the mesorectal fascia upon staging, inter-tuberous distance <10 cm) with mid-to-low, non-metastatic rectal cancer (RESET Study ClinicalTrials.gov Identifier: NCT03574493).

## Conclusion

According to the results of the present study, which investigated a large cohort of patients, differences in the bony structure of the pelvis exist when comparing male and female patients, and surgeons must be aware of these differences and of the shape of the pelvis when approaching the rectum, in particular when using a bottom-up technique. Moreover, the perspective highlighted how these differences might have an impact on the distribution of mesorectum, with a consequence on the surgical planning if a total mesoractal excision is required. Increasing the knowledge on pelvis structure and mesorectum distribution may help also in assessing appropriate patients for a trans-abdominal or trans-anal approach.

## Supplementary information

Supplement Figure 1. PubMed Search March 2019: “TaTME[All Fields]” vs “TaTME[All Fields] AND (“complications”[Subheading] OR “complications”[All Fields])”. (TIFF 33989 kb)

High resolution image (PNG 385 kb)

Supplement Figure 2. Pelvimetry 3D Segmentation. **A.** Automatic Segmentation Process by means of Level Tracing Effect Algorithm: the edge identification (on the left) and filling phase (on the right) are shown; **B.** 3D Only Model View; **C.** Laplacian Filter scheme. The umbrella region grows with increasing filer order; **D.** Comparison of the 3D Model before (a) and after (b) the smoothing process: front view; **E.** Comparison of the 3D Model before (a) and after (b) the smoothing process: side view; **F.** 3D Model with markers. (TIFF 33989 kb)

High resolution image (PNG 2090 kb)
