# Supplementary figures and images for: 3D pelvimetry and biometric measurements: a surgical perspective for colorectal resections
Source: Int J Colorectal Dis. 2020 Nov 23;36(5):977–86. doi: 10.1007/s00384-020-03802-9 (PMC8026460; doi:10.1007/s00384-020-03802-9)

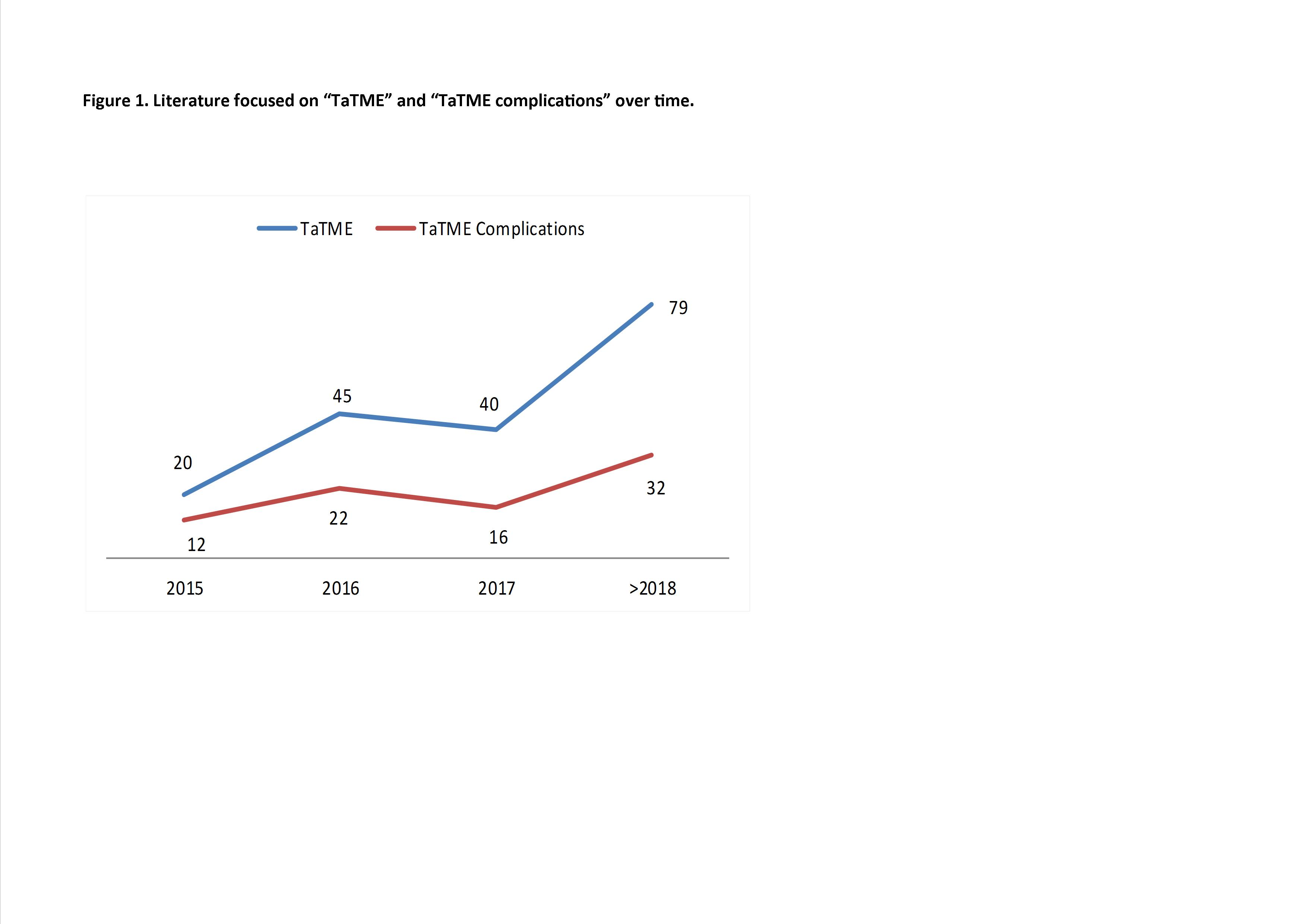

Supplement: Supplementary file 3 — PubMed Search March 2019: “TaTME[All Fields]” vs “TaTME[All Fields] AND (“complications”[Subheading] OR “complications”[All Fields])”. (TIFF 33989 kb) [file 384_2020_3802_MOESM1_ESM.tiff]

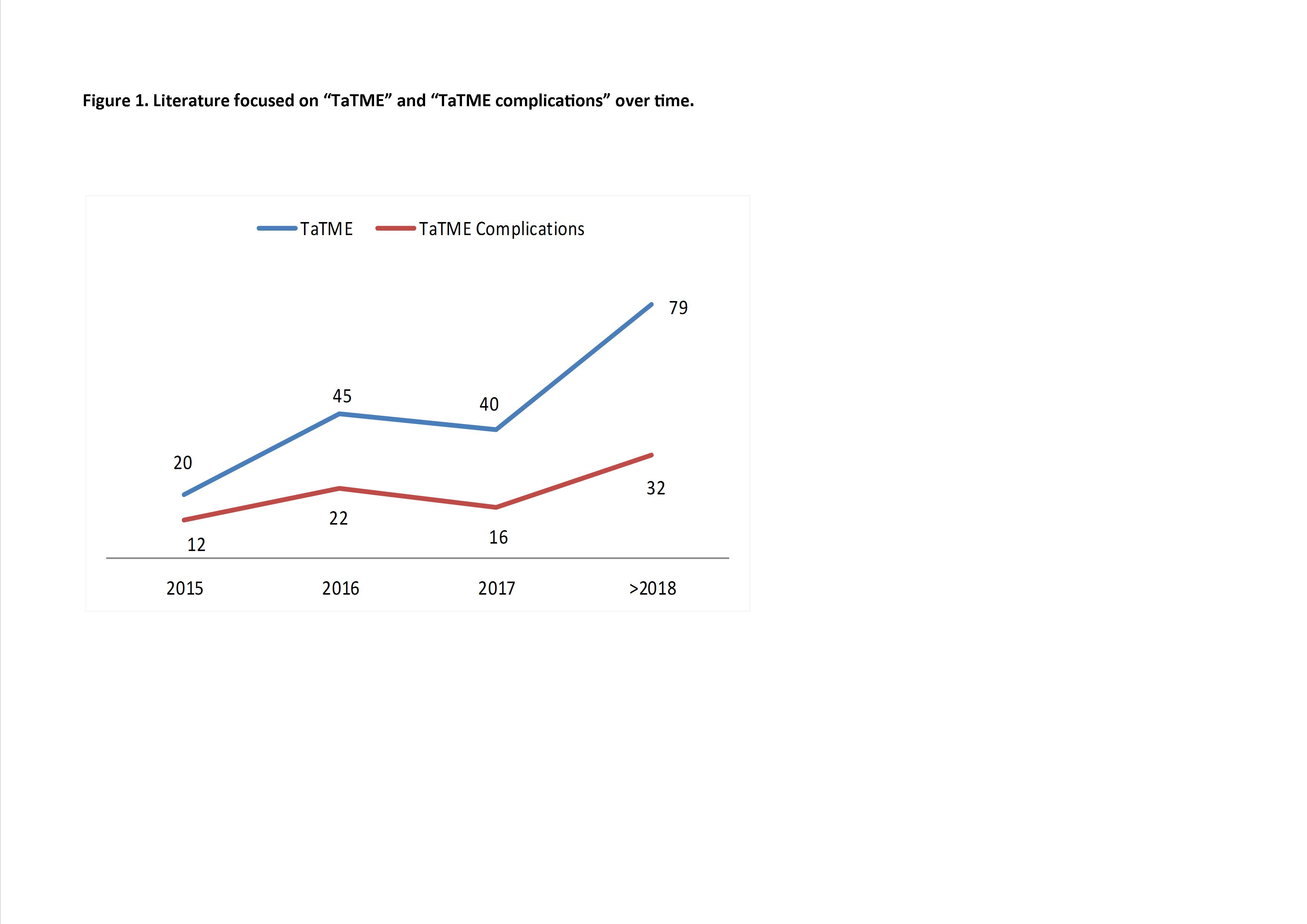

Supplement: Supplementary file 4 — High resolution image (PNG 385 kb) [file 384_2020_3802_Fig5_ESM.png]

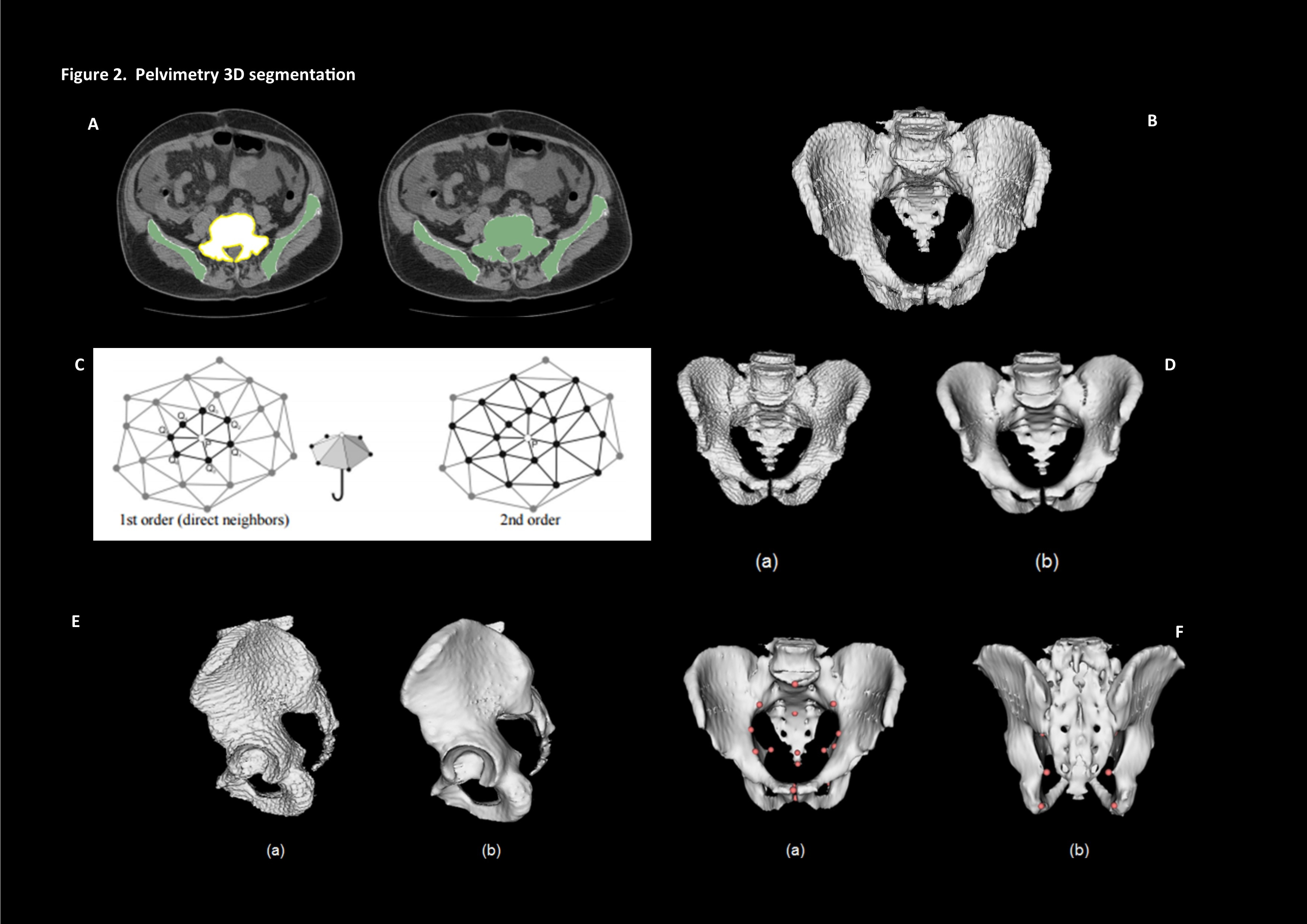

Supplement: Supplementary file 5 — Pelvimetry 3D Segmentation. A. Automatic Segmentation Process by means of Level Tracing Effect Algorithm: the edge identification (on the left) and filling phase (on the right) are shown; B. 3D Only Model View; C. Laplacian Filter scheme. The umbrella region grows with increasing filer order; D. Comparison of the 3D Model before (a) and after (b) the smoothing process: front view; E. Comparison of the 3D Model before (a) and after (b) the smoothing process: side view; F. 3D Model with markers. (TIFF 33989 kb) [file 384_2020_3802_MOESM2_ESM.tiff]

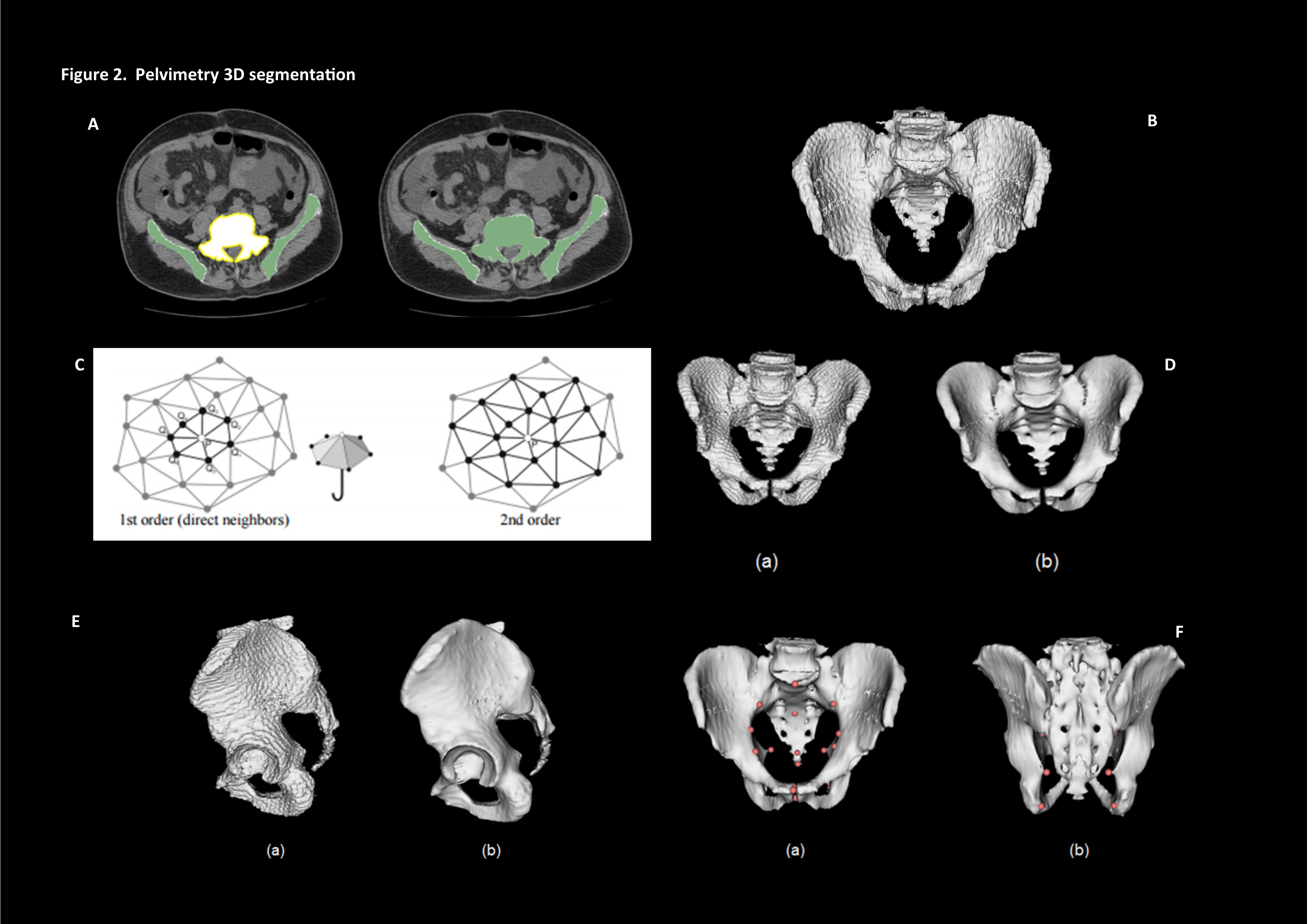

Supplement: Supplementary file 6 — High resolution image (PNG 2090 kb) [file 384_2020_3802_Fig6_ESM.png]
